# Production of Highly Porous Carbon Materials from Coastal Driftwood

**DOI:** 10.3390/ma19173629

**Published:** 2026-08-26

**Authors:** Hervan Marion Morgan, Chi-Hung Tsai, Wen-Tien Tsai

**Affiliations:** 1 Department of Tropical Agriculture and International Cooperation, National Pingtung University of Science and Technology, Neipu Township, Pingtung 912, Taiwan; p11222364@mail.npust.edu.tw; 2 Department of Resources Engineering, National Cheng Kung University, Tainan 701, Taiwan; 3 Graduate Institute of Bioresources, National Pingtung University of Science and Technology, Pingtung 912, Taiwan

**Keywords:** driftwood, pyrolysis, biochar, pore property, elemental analysis

## Abstract

To promote the value-added circular utilization of coastal driftwood, a single salt-exposed driftwood specimen was thermally converted into porous carbon materials by slow pyrolysis. Highly porous carbons are attractive for applications such as adsorption and catalyst support because accessible micro- and mesopores provide a large interfacial area. Prior to carbonization, the thermochemical characteristics of the driftwood were evaluated by proximate analysis, elemental analysis, calorific-value determination, and thermogravimetric analysis (TGA). Pyrolysis was conducted at 400, 500, 600, 700, and 800 °C with residence times of 0, 30, and 60 min at a heating rate of 10 °C/min. The crude biochar products were subsequently washed with reverse-osmosis water. Carbonization temperature was the principal process variable governing pore development. The condition producing the maximum measured porosity was 800 °C with a 60 min residence time, for which the crude biochar yield was 22.98 wt%. The instrument-reported BET surface area and total pore volume of DW-800-60 were 777.5 m^2^/g and 0.47 cm^3^/g, respectively. Re-evaluation of the same N_2_ isotherm using the Rouquerol consistency criteria gave a physically consistent BET estimate of approximately 944.0 m^2^/g over P/P_0_ = 0.0051–0.0597. Gas adsorption indicated a predominantly microporous structure with an additional mesoporous contribution, whereas the scanning electron microscope (SEM) showed inherited micrometer-scale wood channels. DW-800-60 contained 87.6 wt% carbon. Because the botanical species and mineral-salt composition were not identified and practical adsorption or catalytic performance were not tested, the results should be regarded as specific to the investigated specimen and as a basis for future application-oriented evaluation.

## 1. Introduction

Driftwood is woody debris that may originate from natural and/or anthropogenic sources and accumulate in coastal waters, along shorelines, and on riverbanks. In natural environments, driftwood provides rafting substrates, refuges, and habitats for invertebrates, plants, seeds, and other organisms [1]. However, trees, logs, and wood fragments can be mobilized and deposited during severe weather events, especially heavy rainfall and typhoons [2]. Accumulated driftwood can contribute to sediment retention, local erosion, obstruction of waterways, and management burdens along coastlines. As a potentially recoverable coastal biomass resource, it has been reused as a fuel and as a construction material [3,4,5]. Open burning of chlorine-containing driftwood may release hazardous air pollutants, including dioxin-related compounds, and coastal salts can also contribute to slagging and fouling during thermal conversion [6,7,8]. Consequently, water washing has been recommended when the objective is to reduce salt loading before combustion or related thermal utilization. In the present study, however, the feedstock was intentionally carbonized in its unwashed, as-collected condition because pre-pyrolysis washing was not included in the experimental design; washing was instead applied to the resulting biochar before characterization.

Unlike purpose-grown wood, single-species forestry residues, or standardized lignocellulosic feedstocks, coastal driftwood should not be regarded as a homogeneous biomass category. Its thermochemical behavior can vary with factors such as its botanical origin, degree of weathering/degradation, and the amount of retained salts and mineral particles. These factors can alter ash content, devolatilization behavior, biochar yield, and pore development. Alkali and alkaline-earth metals such as Na, K, Ca, and Mg are known to influence lignocellulosic pyrolysis reactions [9], although their effects may depend strongly on the associated anion and feedstock; for example, NaCl and KCl have been reported to exert substantially weaker catalytic effects than carbonate salts [10]. Therefore, the results obtained from an individual coastal specimen should be interpreted as feedstock-specific unless broader sampling and mineral characterization are performed.

Only a limited number of studies have investigated the thermochemical conversion of driftwood into energy, fuels, and carbonaceous materials using processes such as gasification [11,12,13] and pyrolysis [14,15,16]. Regarding driftwood pyrolysis, Tröger et al. [14] investigated fast pyrolysis for the energetic utilization of the resulting products in electricity and heat generation and established a database of biomass pyrolysis experiments. Qatarneh et al. [15] characterized the physicochemical properties of river driftwood, including its moisture content, elemental composition, higher heating value, and lignocellulosic composition (i.e., cellulose, hemicellulose, and lignin). Given its high lignocellulosic content and low ash content [15], river driftwood could be blended and further processed as a feedstock for biochar production through pyrolysis or hydrothermal carbonization, regardless of its botanical origin or type. In a subsequent study, the potential use of river driftwood as a hard-carbon precursor for sodium-ion batteries was investigated [16]. Initially, hydrothermal carbonization (HTC) was carried out at temperatures ranging from 180 to 220 °C to produce hydrochar, followed by an upgrading pyrolysis treatment at 1400 °C under an inert atmosphere to form hard carbon [16]. The resulting hard carbon materials exhibited high Coulombic efficiencies and reversible capacities. However, characterization of their pore structures and chemical compositions revealed relatively low specific surface areas (i.e., 5.0–21.5 m^2^/g) and carbon contents (i.e., 48.9–64.8 wt%, on a dry basis) among the different driftwood samples.

In a previous study [17], the thermochemical properties of river driftwood were evaluated for energy recovery in Taiwan, and the resulting biochar produced at 500 °C exhibited a higher heating value of 25.5 MJ/kg. Building on this background, the present study investigated the physical carbonization of an unwashed, salt-exposed coastal driftwood specimen over a comparatively broad range of temperatures and residence times, without deliberate chemical activation. The objective was to determine how carbonization severity affected char yield, pore characteristics, surface morphology, and elemental composition, and to identify the condition that produced the maximum pore development within the investigated matrix. Nitrogen adsorption–desorption analysis and SEM were used to characterize pore-related properties at different length scales, while elemental analysis was used to evaluate carbon enrichment. Because only one specimen was investigated, the study was not intended to establish universal properties for all coastal driftwood; rather, it provides a feedstock-specific case study and a basis for subsequent work involving multiple driftwood sources, mineral analysis, and application testing.

## 2. Materials and Methods

### 2.1. Driftwood

The starting biomass, coastal driftwood (denoted as DW), was obtained as one large piece from a local beach in Cijin District, Kaohsiung City, Taiwan. The specimen was salt-exposed and weathered, approximately 60 cm in length and 8 cm in diameter. Its botanical species could not be determined, but it may be China tree (*Melia azedarach*) [17]. The specimen retained relatively high density despite weathering and therefore appeared qualitatively hardwood-like; however, this observation was not used as a taxonomic identification. Accordingly, the results reported herein refer specifically to this individual specimen. No water-washing pretreatment was applied before pyrolysis because prewashing was not part of the experimental design. To satisfy the feedstock size requirements of the vertical carbonization reactor, which had an inner diameter of approximately 5 cm, the raw driftwood was chipped, shredded, and sieved to obtain particles ranging from 0.841 mm (No. 20 mesh opening) to 1.70 mm (No. 12 mesh opening), corresponding to an approximate mean particle size of 1.27 mm. Before thermochemical characterization and pyrolysis, the material was dried in an air-circulating oven (Taiwan HIPOINT Co., Kaohsiung city, Taiwan) at 105 °C for at least 12 h to remove residual moisture and loosely adsorbed water.

### 2.2. Determinations of Thermochemical Characteristics of Driftwood

The thermochemical characteristics of the driftwood, including proximate composition, elemental composition, and calorific value, were determined according to procedures described previously [18,19]. Thermogravimetric analysis (TGA) and derivative thermogravimetric analysis (DTG) were performed using a thermal analyzer (Model TGA-51; Shimadzu Co., Tokyo, Japan) from room temperature to 1000 °C under a continuous nitrogen (N_2_) atmosphere at heating rates of 5, 10, 15, and 20 °C/min. The multiple heating rates were used to evaluate the heating-rate dependence of the apparent decomposition temperatures, distinguish kinetic/thermal-lag shifts from the overall decomposition pattern, and select an appropriate heating regime for the subsequent carbonization experiments. A heating rate of 10 °C/min was then used for all furnace experiments to provide a slow-pyrolysis condition while remaining consistent with the TGA comparison. The as-received driftwood was used for proximate analysis. The higher heating value (HHV) of the dried driftwood was determined using an adiabatic calorimeter (Model 6200; Parr Instrument Company, Moline, IL, USA), and its elemental composition was determined using an elemental analyzer (vario EL cube; Elementar GmbH, Langenselbold, Germany).

### 2.3. Carbonization Experiments

The carbonization experiments were carried out in a vertical furnace (One Home Drying Machinery Co., Kaohsiung city, Taiwan) according to procedures used in previous studies [18,19]. Approximately 5 g of dried driftwood was heated from room temperature to nominal carbonization temperatures of 400, 500, 600, 700, or 800 °C at 10 °C/min under a continuous N_2_ flow of 500 cm^3^/min. The reactor-zone temperature was monitored by a thermocouple positioned directly adjacent to the sealed pyrolysis tube/chamber; therefore, the reported temperatures represent the monitored thermal environment of the sample rather than a thermocouple embedded within individual particles. Residence times were 0, 30, or 60 min. A residence time of 0 min was defined as termination of furnace power immediately after the monitored reactor zone reached the target temperature, with no intentional isothermal holding period. After each run, the furnace was opened and rapid external cooling was assisted by a high-speed fan while N_2_ flow was maintained to preserve an oxygen-deficient atmosphere. The sealed chamber was not opened until the temperature had decreased to approximately 60-70 °C to avoid oxidation or combustion of the hot char. The crude biochar yield was calculated from the mass of biochar before washing relative to the initial dry driftwood mass. The pyrolysis/yield screening matrix was not independently replicated; the reported yields are therefore single-run values. For post-pyrolysis cleaning, each crude char was washed once at 25 °C with laboratory reverse-osmosis water at a solid-to-liquid ratio of 1:50 (*w*/*v*) under magnetic stirring for 1 h at 60 rpm. The biochar was then allowed to settle, the wash water was decanted, and the solid was further dried at 105 °C before subsequent use. Washed-product mass was not recorded because the determination of washed yield was not included in the original experimental design; all yield values in this article consequently refer to crude, prewashing biochar. Sample codes indicate feedstock, temperature, and residence time; for example, DW-800-60 denotes driftwood-derived biochar produced at 800 °C with a 60 min residence time.

### 2.4. Determinations of Pore and Chemical Characteristics of DW-Derived Biochar

The pore characteristics of the DW-derived biochar products were analyzed using an automated adsorption–desorption analyzer (ASAP 2020 Plus; Micromeritics Instrument Corporation, Norcross, GA, USA). Before N_2_ adsorption–desorption analysis at −196 °C, samples were degassed under vacuum for a total of approximately 24 h: the temperature was first increased from room temperature to 90 °C and held for 1 h, then increased to 200 °C at 5 °C/min and maintained at 200 °C for the remainder of the degassing period. The originally generated screening BET values were calculated by the instrument workflow using 15 adsorption points in the nominal P/P_0_ range of approximately 0.05-0.30. Because a fixed range can be inappropriate for strongly microporous carbons, the raw DW-800-60 isotherm was additionally re-evaluated according to the Rouquerol consistency criteria [20]. A physically consistent interval of P/P_0_ = 0.0051–0.0597 (seven points) gave a positive BET constant (C ≈ 1.21 × 10^3^), a monolayer relative pressure of approximately 0.028 within the selected interval, R^2^ = 0.99998, and a reassessed BET area of approximately 944 m^2^/g. The instrument-reported screening value (777.54 m^2^/g) is retained (seen in Table 2 of Section 3.2) to preserve comparability across the complete experimental matrix, while the Rouquerol-consistent reassessment is used to demonstrate that the conclusion of unusually high pore development for DW-800-60 is robust. Total pore volume was estimated from N_2_ uptake near P/P_0_ ≈ 0.995, and micropore area/volume was obtained by the *t*-plot method where physically meaningful. Mesopore properties were evaluated using the Barrett–Joyner–Halenda (BJH) method, whereas the Horváth–Kawazoe (HK) method was used as a model-dependent estimate of the micropore-size distribution [21]. The NLDFT/QSDFT pore-size analysis was not available in the archived analytical workflow and is therefore identified as a recommended refinement for future work. Surface morphology was examined by SEM (S-3000N; Hitachi High-Tech Corporation, Tokyo, Japan) at 15 keV; SEM imaging was performed for DW-800-60, and localized EDS analysis of the bright surface particles was not performed. The C, H, N, and S contents of the biochar products were determined in duplicate by elemental analysis. Means and standard deviations are reported in Table 3. Oxygen was calculated by difference as an apparent value, O* = 100 - C - H - N - S; because post-pyrolysis ash/mineral content was not independently quantified, O* may include an unmeasured inorganic contribution and should not be interpreted as ash-corrected oxygen.

## 3. Results and Discussion

### 3.1. Thermochemical Characteristics of Coastal Driftwood

Table 1 summarizes the thermochemical properties of the investigated coastal driftwood specimen. The feedstock exhibited a carbon content of 39.33 wt% (dry basis), a high volatile-matter content of 78.40 wt% (as-received basis), and relatively low nitrogen and sulfur contents of 1.32 and 0.29 wt%, respectively. Its ash content was 3.91 wt% on an as-received basis, which is higher than that of many clean woody biomasses and is consistent with the possibility of retained coastal mineral matter [22]. However, individual inorganic species (e.g., Na, Cl, Ca, or Mg) were not quantified in this study, and their concentrations should therefore not be inferred from the bulk ash value [23]. The HHV was 17.87 MJ/kg (dry basis). From the elemental composition, the approximate atomic H/C and apparent O/C ratios of the feedstock were 1.56 and 1.03, respectively, which are more directly interpretable for biomass carbonization than assigning a pseudo-molecular formula to a heterogeneous lignocellulosic material. Taken together, the proximate and elemental characteristics indicate that this individual driftwood specimen can be carbonized to produce a carbon-rich solid, although its behavior should not be generalized to coastal driftwood of different botanical or exposure histories [15].

**Figure 1:**
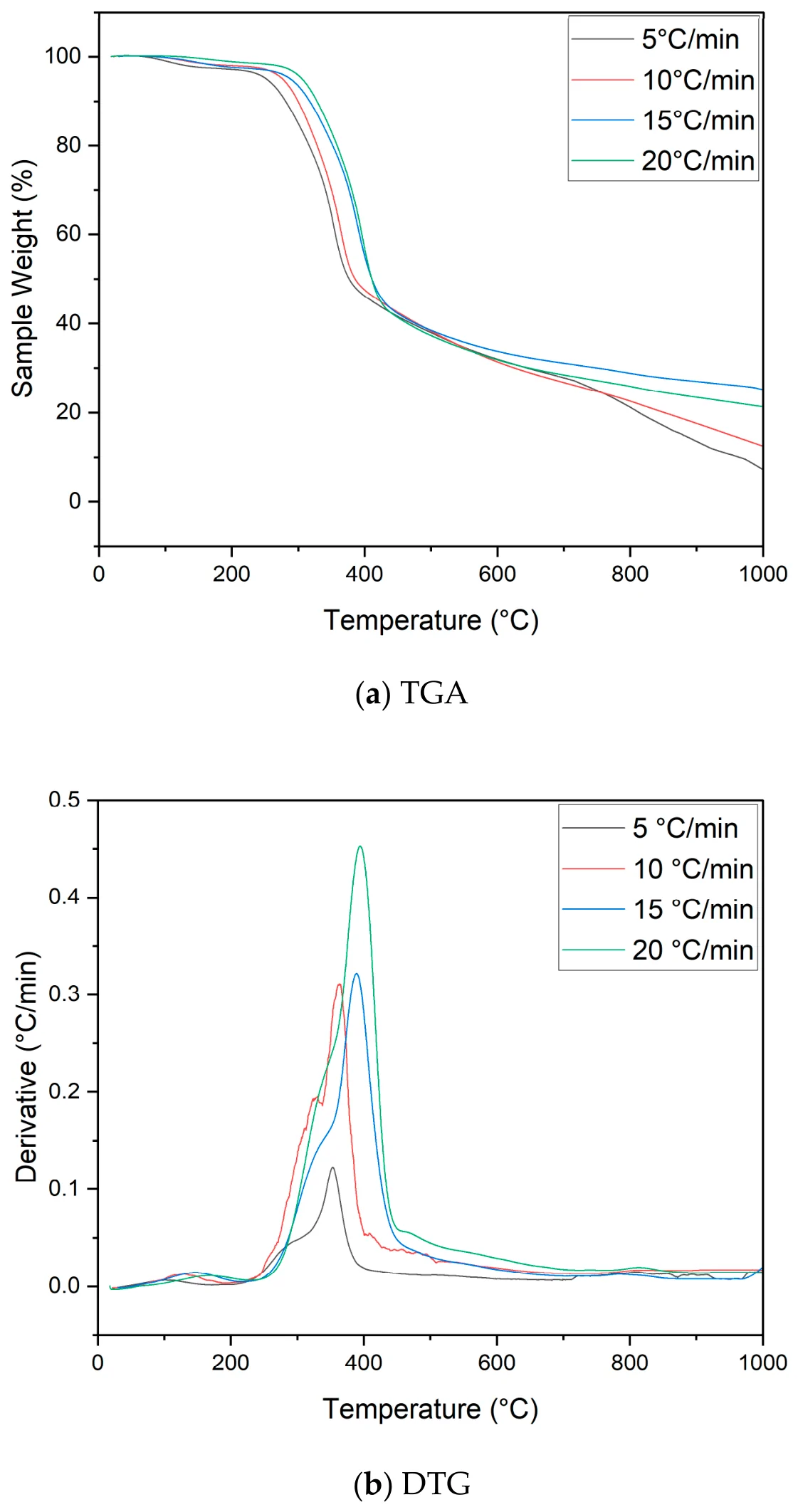
(**a**) Thermogravimetric analysis (TGA) curves of coastal driftwood and (**b**) its derivative thermogravimetry (DTG) curves at the heating rates of 5, 10, 15 and 20 °C/min.

Thermogravimetric analysis (TGA) provides a rapid means of assessing the thermal decomposition of a lignocellulosic material under a controlled atmosphere. Figure 1a shows the TGA curves obtained at 5, 10, 15, and 20 °C/min under N_2_, and Figure 1b shows the corresponding DTG profiles. The overall decomposition pattern remained similar at all heating rates, whereas the apparent decomposition features shifted to higher temperatures as the heating rate increased. Such shifts are commonly attributed to a combination of finite heat/mass transfer and the reduced time available for decomposition at a given programmed temperature [24]. The first region, approximately 50–200 °C, was dominated by moisture removal and release of light volatiles. The major mass-loss region occurred from approximately 200 to 400–450 °C. Hemicellulose, which is relatively thermally labile and structurally heterogeneous, contributed to the lower-temperature shoulder, while cellulose decomposition contributed strongly to the principal DTG maximum; lignin decomposed over a broader temperature interval and therefore contributed to the extended tail rather than a single narrow peak [22,24]. The approximately 50 wt% loss through this region reflected extensive devolatilization and explained why the subsequently measured char yields decreased as the furnace temperature was increased. Above approximately 400–450 °C, further lignin degradation, secondary cracking, condensation, and aromatization progressively enriched the solid in carbon while additional volatile release reduced solid yield. These observations supported selection of 400–800 °C for the carbonization matrix and also explained the qualitative agreement between the TGA residual masses and the furnace-derived crude biochar yields.

### 3.2. Yields and Pore Properties of DW-Derived Biochar Materials

**Figure 2:**
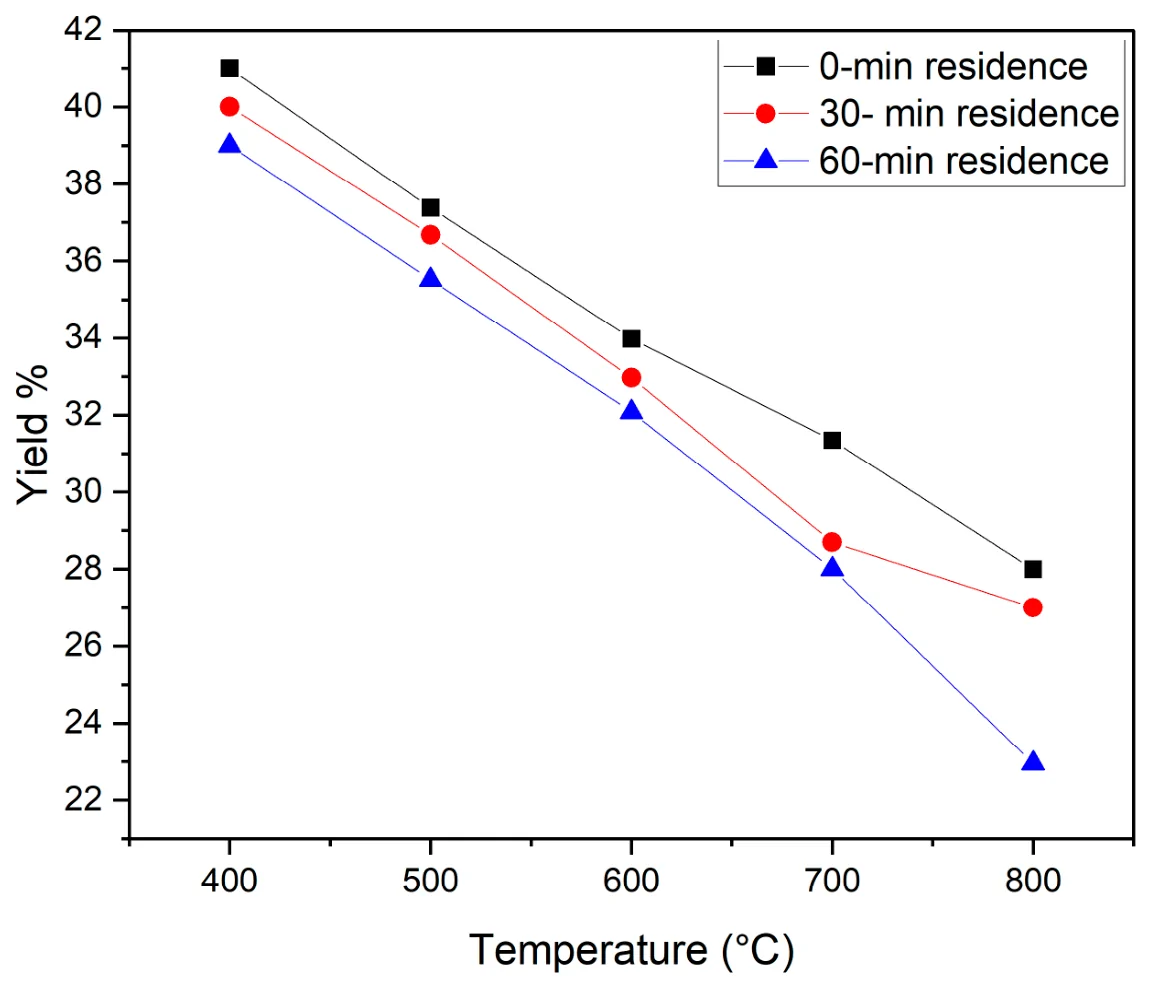
Variations in yields of resulting biochar materials at different pyrolysis conditions.

As shown in Figure 2 and summarized numerically in Table 2, the crude yield of DW-derived biochar generally decreased as carbonization temperature increased from 400 to 800 °C, whereas the additional decrease associated with residence time was smaller. For the 60 min series, the yield decreased from 39.91 wt% at 400 °C to 22.98 wt% at 800 °C. At 800 °C, the yield decreased from 27.21 wt% at 0 min to 22.98 wt% at 60 min. These values were broadly consistent with the TGA residual-mass trend at the same 10 °C/min heating rate. Increasing the thermal severity promoted the devolatilization and cracking of the remaining lignocellulosic and oxygenated structures, releasing CO, CO_2_, H_2_, CH_4_, water, and condensable organics while enriching the residual carbonaceous matrix [25]. The simultaneous increase in N_2_-accessible surface area at higher temperatures (Table 2) confirmed that the observed pore development was not inferred solely from SEM morphology. Nevertheless, 800 °C/60 min also produced the lowest yield and required the greatest thermal severity in the investigated matrix. It was therefore described hereafter as the condition producing the maximum measured surface area, rather than as a technological or economic optimum.

The principal pore properties of the DW-derived biochar products and their crude yields are summarized in Table 2. For DW-400-60, the low-uptake analysis produced a negative BET constant and a negative *t*-plot external area, causing the calculated micropore area to exceed the BET area and the micropore volume to exceed the total pore volume. Those nonphysical derived quantities are therefore designated as not reliable (n.r.) rather than reported as valid pore properties. This limitation is expected when N_2_ uptake is extremely small and illustrates why derived pore-size metrics for very low-surface-area chars must be interpreted cautiously. Based on this, the effects of the process temperatures and residence time on the pore properties are discussed further.

**Figure 3:**
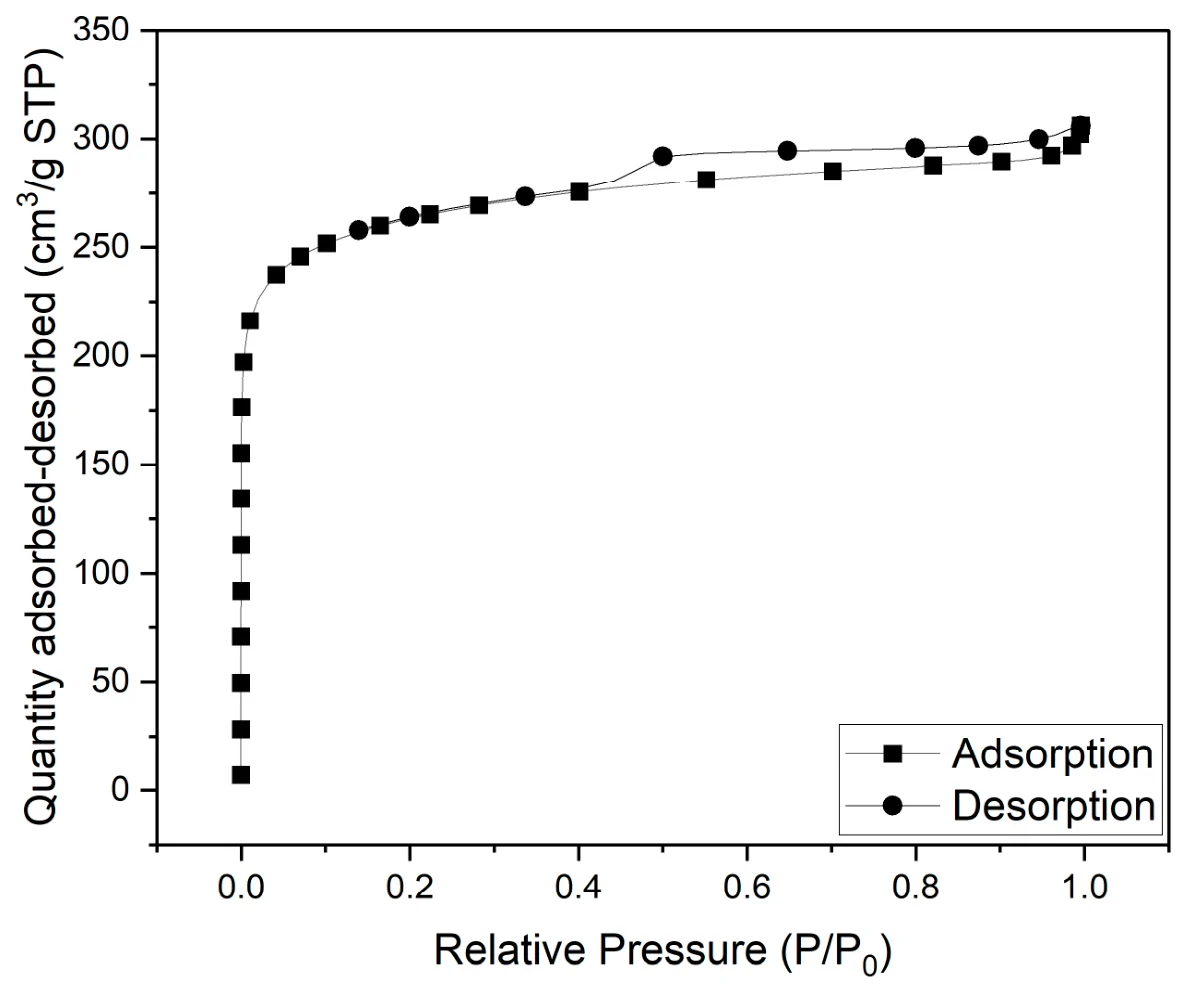
N_2_ adsorption–desorption isotherms of maximum surface area biochar product (DW-800-60).

**Figure 4:**
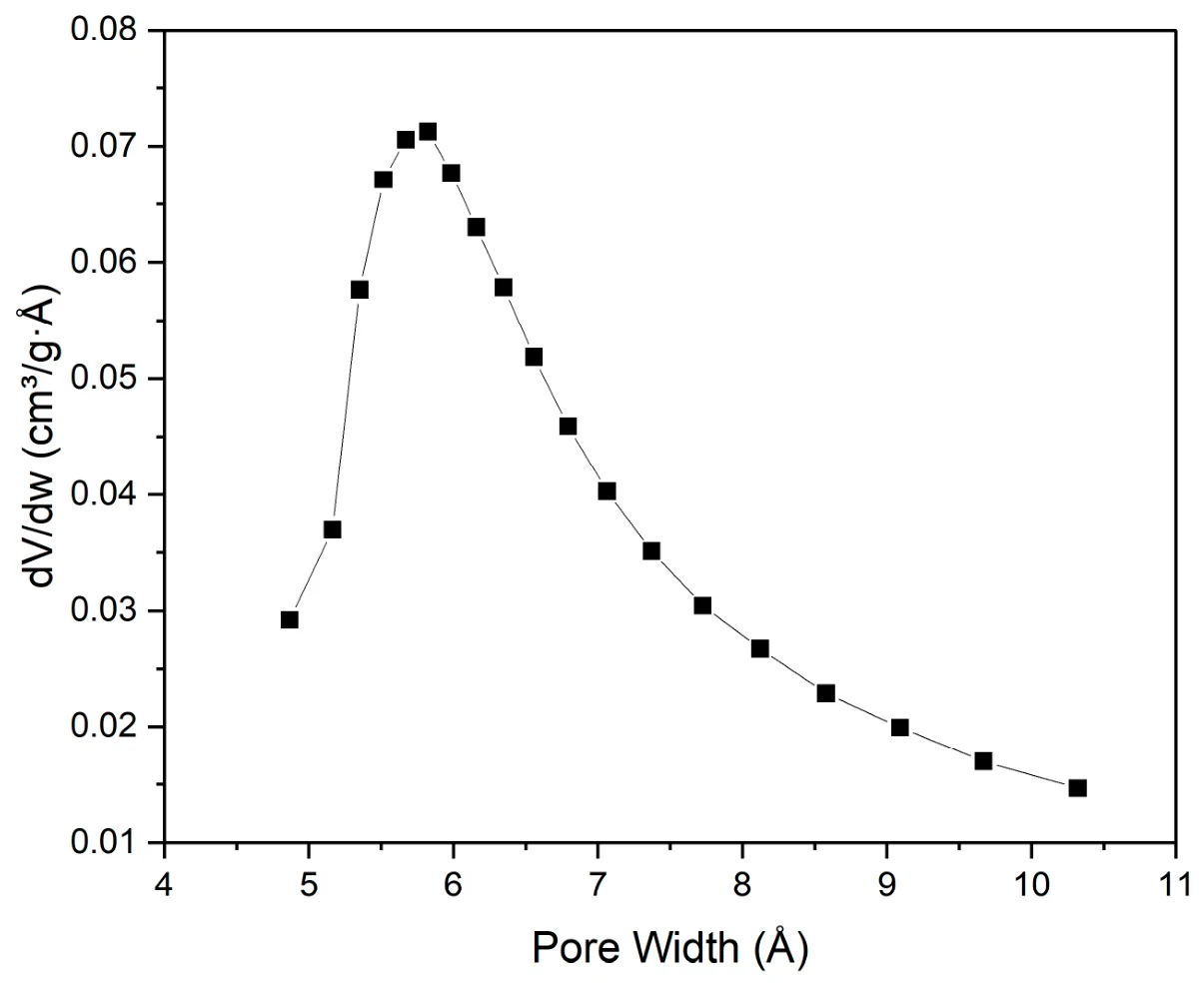
HK-derived micropore size distribution of the maximum-surface-area biochar product (DW-800-60).

**Figure 5:**
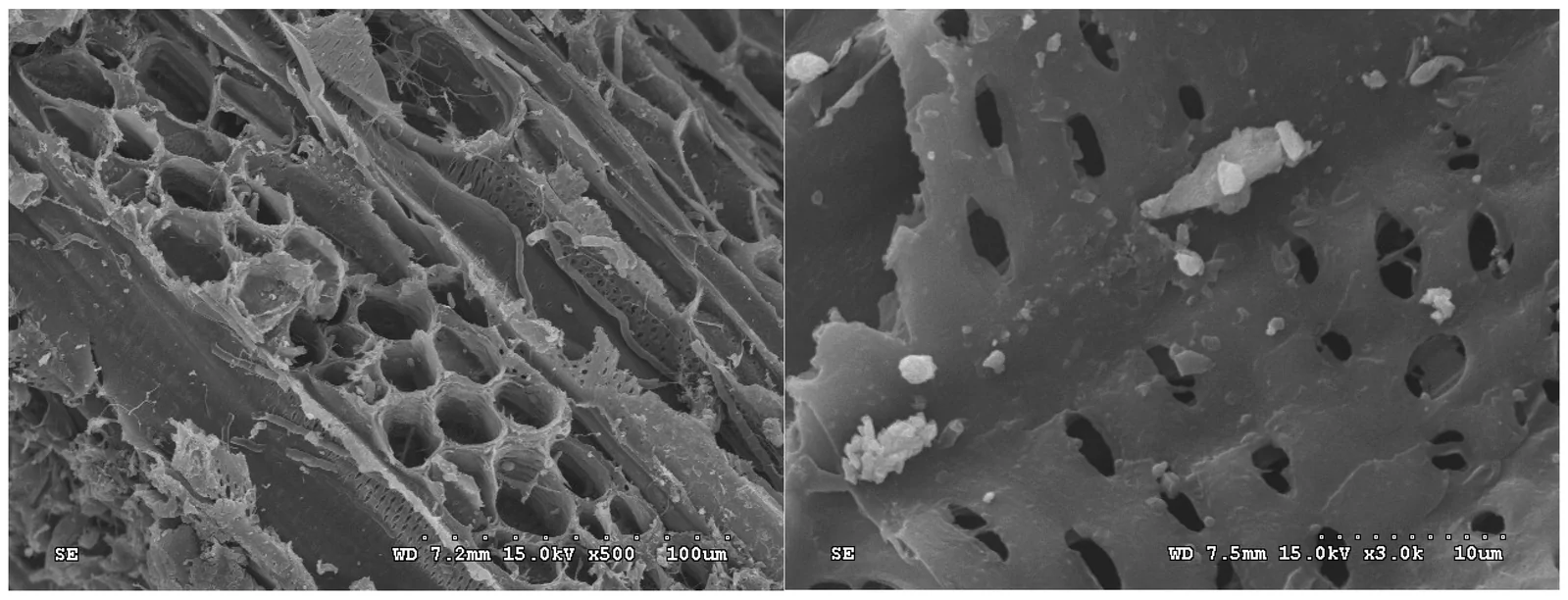
SEM images (**Left:** ×500; **Right:** ×3000) of DW-800-60. The visible channels are micrometer-scale morphological features and do not directly resolve BET microporosity.

1. Regarding temperature, marked pore development occurred between 500 and 600 °C at all three residence times. For example, instrument-reported BET areas increased from approximately 1–6 m^2^/g at 500 °C to approximately 123–306 m^2^/g at 600 °C, depending on residence time. Further increases were observed at 700 and 800 °C. These data indicated that carbonization temperature exerted a stronger influence on accessible pore development than residence time within the investigated ranges. The trend was consistent with intensified devolatilization, secondary cracking, and progressive opening of previously inaccessible voids as thermal severity increased [26,27]. At the same time, increasing pore development was accompanied by declining crude yield, emphasizing the trade-off between carbon recovery and porosity.
2. Residence time also influenced pore development, particularly at the higher carbonization temperatures, although its effect was generally secondary to that of temperature. At 800 °C, the instrument-reported BET area increased from 413 m^2^/g at 0 min to 590 m^2^/g at 30 min and 778 m^2^/g at 60 min. The associated total pore volume increased from 0.23 to 0.34 and 0.47 cm^3^/g, respectively. Continued devolatilization and secondary cracking during the isothermal period likely promoted additional opening and widening of pores. However, these improvements occurred together with additional mass loss; consequently, extending residence time cannot be evaluated solely on the basis of surface area.
3. Figure 3 presents the N_2_ adsorption–desorption isotherm for DW-800-60, which was selected for detailed presentation because it exhibited the highest pore development among the instrument-screened samples. The steep uptake at very low relative pressure is characteristic of substantial micropore filling, while the additional uptake and adsorption–desorption separation at intermediate-to-high relative pressure indicate that the material is not adequately described as a purely Type I microporous solid [28]. It is therefore interpreted more cautiously as a Type I-dominated micro/mesoporous carbon. The screening *t*-plot result assigned 516.0 m^2^/g of the 777.5 m^2^/g instrument-reported BET area to micropore area, and the total pore volume was 0.47 cm^3^/g. Importantly, application of the original fixed BET window to DW-800-60 produced a negative BET constant, demonstrating that a high linear correlation coefficient alone was insufficient to establish physical validity. Reanalysis using the Rouquerol criteria [20] over P/P_0_ = 0.0051–0.0597 yielded C ≈ 1.21 × 10^3^, a monolayer relative pressure of approximately 0.028 within the selected range, R^2^ = 0.99998, and a BET estimate of approximately 944.0 m^2^/g. Thus, while the absolute BET value is method-dependent for this strongly microporous carbon, both analyses support the conclusion that DW-800-60 developed an unusually high N_2_-accessible surface area without deliberate chemical activation. Figure 4 shows an HK median pore width of approximately 0.568 nm; this value should be regarded as a model-dependent estimate because the HK calculations require idealized pore-geometry and adsorption-potential assumptions. The NLDFT or QSDFT using an appropriate carbon slit-pore model would provide a more rigorous pore-size distribution and is recommended for future analysis.
4. The high surface area obtained by physical pyrolysis alone warranted additional consideration. Severe devolatilization at 800 °C, combined with the pre-existing vascular architecture of wood and progressive carbon-matrix contraction, can create and open a large micropore network. In addition, the as-received feedstock contained 3.91 wt% ash, suggesting that mineral matter was present. Alkali and alkaline-earth species can modify biomass pyrolysis pathways and char development [9], the study by De Smedt et al. [29] demonstrated that a ZnCl_2_-NaCl-KCl molten-salt mixture can strongly develop porosity during activated-carbon production. However, NaCl itself should not be assumed to have acted as an activating agent in the present study: recent work found that NaCl and KCl alone had comparatively small effects on pyrolysis products relative to carbonate salts [10]. Because Na, Cl, Ca, Mg, and related species were not quantified before and after washing, any contribution of inherent coastal salts to pore development remains a plausible but unverified mechanism rather than a demonstrated cause.
5. Scanning electron microscopy was performed on DW-800-60 to examine the surface morphology and micrometer-scale structure (Figure 5). The images reveal elongated channels and cavities that are consistent with retention of the original wood vascular architecture after carbonization. These micrometer-scale features should not be interpreted as direct evidence of the microporosity responsible for the BET/*t*-plot results, because most micro- and small mesopores are below the resolution represented in the presented SEM fields. The brighter particles visible in the higher-magnification image may represent residual inorganic matter or other surface deposits, but localized EDS was not performed on these particles; consequently, they cannot be assigned specifically to NaCl or any other mineral phase.

### 3.3. Elemental Analysis of DW-Derived Biochar Materials

The elemental compositions of the DW-derived biochar products were evaluated from duplicate C, H, N, and S measurements, with oxygen reported as the apparent difference value O* = 100 - C - H - N - S. Table 3 reports the replicate mean ± standard deviation for the measured elements and explicitly calculated molar H/C and apparent O/C ratios. Because ash was not measured for each washed biochar, O* and the corresponding O/C ratio may be overestimated by any residual inorganic fraction and should therefore be interpreted as approximate compositional indicators rather than ash-corrected oxygen values.

O* = 100 - C - H - N - S

Table 3 shows that carbon enrichment generally increased with carbonization severity, while hydrogen and apparent oxygen decreased. For the 60 min series, mean carbon content increased from 64.19 wt% at 400 °C to 87.60 wt% at 800 °C, whereas mean hydrogen decreased from 3.42 to 1.51 wt% and apparent oxygen decreased from 29.74 to 7.70 wt%. These trends were consistent with those reported for bamboo-derived biochar products [19] and other lignocellulosic feedstocks [30,31]. The duplicate measurements also revealed appreciable analytical/sample heterogeneity for several carbon determinations, which is represented by the standard deviations. The highest valid carbon value was obtained for DW-800-60. For this sample, one archived carbon content exceeded 100 wt% and was therefore physically invalid; that result was excluded, so the reported DW-800-60 carbon content is based on the remaining valid carbon determination, while H, N, and S remain duplicate means.

The compositional trends were consistent with progressive devolatilization, cracking, condensation, and aromatization of the carbonaceous matrix as the temperature and residence time increased. The explicitly calculated atomic ratios provided a more appropriate measure of this progression than mass-percentage ratios. H/C decreased systematically with increasing temperature within each residence-time series, reaching approximately 0.21 for DW-800-60. Apparent O/C also generally decreased with increasing thermal severity, reaching approximately 0.07 for DW-800-60; however, the 0 min series was not strictly monotonic because O/C increased locally from approximately 0.17 at 600 °C to 0.22 at 700 °C before decreasing to 0.15 at 800 °C. In addition, because O* included any unmeasured mineral/ash contribution, the O/C values were best used to describe the relative rather than the absolute oxygen depletion.

### 3.4. Practical Relevance and Study Limitations

High specific surface area and accessible pore volume can be advantageous for adsorption and catalyst-support applications, but these textural properties alone do not establish practical performance. Surface chemistry, pore accessibility relative to the target molecule, solution chemistry, and regeneration behavior can be equally important. No adsorption or catalytic experiment was included in the present study because application testing was outside the original experimental scope. To place the measured pore properties in context without implying untested performance, Table 4 compares DW-800-60 with recent waste-derived porous carbons for which practical dye adsorption was experimentally demonstrated.

The comparison confirmed that the pore development of DW-800-60 is notably relative to several recently reported waste-derived adsorbents, but it should not be interpreted as evidence that its adsorption capacity would be equal to or exceed those materials. The present study also had several limitations that defined the next experimental steps: only one driftwood specimen of unknown species was used; pre- and post-washing mineral compositions were not measured; washed-product yield was not recorded; the pyrolysis/yield and BET screening was not replicated; and application performance was not tested. Moreover, 800 °C/60 min gave both the maximum measured surface area and the lowest crude yield, and it would be expected to impose the highest energy demand among the studied conditions. A technological optimum would therefore have required replicated experiments and a combined evaluation of yield, energy consumption, processing cost, material performance, and feedstock variability.

## 4. Conclusions

This study demonstrates that the investigated salt-exposed coastal driftwood specimen can be converted by slow physical pyrolysis into a highly porous carbon material, while also showing that the result must be interpreted within the limits of a single, botanically unidentified feedstock. Across 400–800 °C and residence times of 0–60 min, carbonization temperature exerted the strongest influence on crude yield, pore development, and carbon enrichment. The condition producing the maximum measured pore development was 800 °C with a 60 min residence time. DW-800-60 had a crude prewashing yield of 22.98 wt%, an instrument-reported BET surface area of 777.5 m^2^/g, a total pore volume of 0.47 cm^3^/g, and a carbon content of 87.6 wt%. A Rouquerol-consistent reassessment of its N_2_ isotherm yielded a BET estimate of approximately 944.0 m^2^/g, supporting the conclusion of unusually high N_2_-accessible porosity while also demonstrating the method dependence of BET analysis for strongly microporous carbons. The isotherm was interpreted as Type I-dominated with an additional mesoporous contribution, whereas SEM primarily revealed micrometer-scale channels inherited from the wood structure. The possible role of inherent coastal salts in pore development remains unverified because mineral species were not quantified. Importantly, 800 °C/60 min should not be considered a technological optimum because it combined the highest surface area with the lowest yield and greatest thermal severity. Future work should evaluate multiple driftwood sources, quantify minerals before and after washing, establish washed mass balances and independent reproducibility, and experimentally test adsorption or catalytic performance before practical environmental applications are claimed.

## Figures and Tables

**Table 1:** Thermochemical properties of coastal driftwood.

Property	Value
Proximate analysis ^a, b^	
Ash (wt%)	3.91 ± 0.70
Volatile matter (wt%)	78.40 ± 0.33
Moisture (wt%)	14.66 ± 0.41
Fixed carbon ^c^ (wt.%)	3.04
Elemental analysis ^b, d^	
Carbon (wt%)	39.33
Hydrogen (wt%)	5.16
Nitrogen (wt%)	1.32
Sulfur (wt%)	0.29
Oxygen ^c^ (wt%)	53.90
Calorific value (MJ/kg) ^b, d^	17.87 ± 0.38

^a^ Air-dry basis (as received sample). ^b^ The mean ± standard deviation for three determinations. ^c^ By difference. ^d^ Dry basis.

**Table 2:** Pore properties of and crude yields of DW-derived biochar products.

Biochar Products ^a^	S_BET_^b^ (m^2^/g)	V_t_^c^ (cm^3^/g)	S_mic_^d^ (m^2^/g)	V_mic_^d^ (cm^3^/g)	D_ave_^e^ (nm)	Yield ^f^ (wt%)
DW-400-00	0.9	0.0024	0.1	0.0002	10.51	40.73
DW-500-00	1.4	0.0034	0.2	0.0002	10.20	37.38
DW-600-00	122.8	0.16	34.6	0.0444	5.21	33.98
DW-700-00	282.4	0.16	240.6	0.1250	2.27	31.35
DW-800-00	413.0	0.23	347.4	0.1760	2.24	27.21
DW-400-30	1.6	0.0043	0.7	0.0004	10.83	40.58
DW-500-30	2.3	0.0033	2.3	0.0012	5.87	36.68
DW-600-30	240.9	0.14	200.9	0.10	2.28	32.96
DW-700-30	427.9	0.24	344.9	0.17	2.26	28.70
DW-800-30	590.2	0.34	463.7	0.24	2.31	27.30
DW-400-60	0.2	0.0013	n.r	n.r	n.r	39.91
DW-500-60	6.3	0.0017	3.5	0.0017	10.68	35.51
DW-600-60	306.0	0.17	256.4	0.13	2.17	32.91
DW-700-60	457.6	0.28	341.0	0.17	2.44	28.49
DW-800-60	777.5	0.47	516.0	0.27	2.42	22.98

^a^ Sample notation identifies dried driftwood (DW), carbonization temperature (400–800 °C), and residence time (0–60 min). ^b^ S_BET_ values are the instrument-reported screening values used for comparison across the complete matrix. For DW-800-60, the Rouquerol-consistent reanalysis of the raw isotherm gave approximately 944.0 m^2^/g (P/P_0_ = 0.0051–0.0597; C ≈ 1.21 × 10^3^; R^2^ = 0.99998). ^c^ Total pore volume (V_t_) was obtained from high-relative-pressure N_2_ adsorption near P/P_0_ ≈ 0.995. ^d^ Micropore surface area (S_mic_) and micropore volume (V_mic_) were estimated by the *t*-plot method where the analysis was physically meaningful. ^e^ Average pore diameter (D_ave_) was calculated as 4V_t_/S_BET_. For extremely low-area samples, this ratio is highly unstable and should not be interpreted as a direct pore-size measurement. ^f^ Yield refers to the crude biochar mass before washing. n.r. = not reliable because the DW-400-60 BET/*t*-plot solution was nonphysical (negative BET C and negative *t*-plot external area).

**Table 3:** Elemental compositions and atomic ratios of DW-derived biochar products.

Biochar Product	Carbon ^a^ (wt%)	Hydrogen ^a^ (wt%)	Nitrogen ^a^ (wt%)	Sulfur ^a^ (wt%)	Oxygen* ^a^ (wt%)	H/C (Atomic Ratio)	O/C* (Atomic Ratio)
DW-400-00	60.41 ± 6.02	3.44 ± 0.44	2.45 ± 0.02	0.14 ± 0.05	33.57	0.68	0.42
DW-400-30	63.39 ± 7.76	3.52 ± 0.43	2.66 ± 0.11	0.16 ± 0.01	30.27	0.66	0.36
DW-400-60	64.19 ± 2.29	3.42 ± 0.15	2.52 ± 0.20	0.13 ± 0.00	29.74	0.63	0.35
DW-500-00	69.57 ± 6.63	3.18 ± 0.35	2.58 ± 0.01	0.18 ± 0.01	24.48	0.55	0.26
DW-500-30	74.32 ± 1.61	3.12 ± 0.05	2.79 ± 0.12	0.21 ± 0.03	19.56	0.50	0.20
DW-500-60	68.13 ± 6.97	2.76 ± 0.32	2.46 ± 0.08	0.16 ± 0.04	26.49	0.48	0.29
DW-600-00	76.88 ± 7.46	2.66 ± 0.24	2.61 ± 0.14	0.18 ± 0.02	17.66	0.41	0.17
DW-600-30	76.09 ± 1.17	2.19 ± 0.00	2.54 ± 0.31	0.23 ± 0.01	18.96	0.34	0.19
DW-600-60	76.97 ± 4.13	2.02 ± 0.13	2.52 ± 0.35	0.21 ± 0.02	18.27	0.31	0.18
DW-700-00	74.15 ± 5.60	1.77 ± 0.13	2.41 ± 0.11	0.19 ± 0.03	21.49	0.29	0.22
DW-700-30	78.25 ± 6.41	1.56 ± 0.17	2.55 ± 0.02	0.29 ± 0.04	17.35	0.24	0.17
DW-700-60	79.42 ± 6.75	1.62 ± 0.20	2.33 ± 0.07	0.30 ± 0.03	16.33	0.24	0.15
DW-800-00	79.89 ± 7.91	1.52 ± 0.08	2.42 ± 0.19	0.28 ± 0.02	15.91	0.23	0.15
DW-800-30	82.47 ± 7.88	1.43 ± 0.01	2.84 ± 0.00	0.41 ± 0.08	12.85	0.21	0.12
DW-800-60	87.60 ^b^	1.51 ± 0.32	2.66 ± 0.11	0.53 ± 0.06	7.70	0.21	0.07

^a^ Measured Carbon, Hydrogen, Nitrogen, and Sulfur values are mean ± sample standard deviation of duplicate determinations unless noted. Oxygen* was calculated by difference from the mean CHNS values and was not corrected for residual ash/mineral matter. ^b^ For DW-800-60, one archived carbon content (>100 wt%) was physically invalid and excluded; the reported carbon value is therefore based on one valid determination, whereas Hydrogen, Nitrogen, and Sulfur are duplicate means. H/C and O/C* are molar atomic ratios calculated from the mean composition.

**Table 4:** Comparison with recent waste-derived porous carbons used in practical adsorption studies.

Material/Feedstock	Preparation	S_BET_ (m^2^/g)	Reported Pore Size	Demonstrated Application	Ref.
DW-800-60 (this study)	Physical pyrolysis, 800 °C, 60 min;no deliberate chemical activation	777.5 screening; ~944.0 Rouquerol	D_ave_ 2.42 nm; HK median 0.568 nm (model-dependent)	Not tested; application validation required	This work
*Auricularia auricula* spent mushroom substrate	Direct carbonization, 500 °C (for 2 hr)	341.1	Mesopore development reported	Methylene blue, 28.1 mg/g	[32]
Waste wood-chip porous carbon	Direct carbonization with Fe(NO_3_)_3_ catalyst	up to 435.2	2.197-10.63 nm	Methylene blue, 321.7 mg/g	[33]
Fe^0^/tea-residue biochar composite	Pyrolysis + NaOH activation	382.66	Average 4.97 nm	Methylene blue, 452.5 mg/g	[34]

## Data Availability

The original contributions presented in this study are included in the article. Further inquiries can be directed to the corresponding authors.
